# Supplementary material for: Are carbon emissions trading and green financial instruments synergistic? -Comprehensive quantitative research based on content analysis
Source: PLoS One. 2024 Mar 7;19(3):e0298601. doi: 10.1371/journal.pone.0298601 (PMC10919601; doi:10.1371/journal.pone.0298601)
Supplement: S1 Data — (ZIP) [file pone.0298601.s001.zip › Data/地级市Z_F工作报告.zip!/─┌├╔╣┼/═¿┴╔╩╨/└┤╫╘╣1⁄2╓┌║┼í╛┬φ┐╦╩2╛▌═°í┐_2019.docx]

各位代表：

现在，我代表市人民政府向大会报告工作，请予审议，并请市政协各位委员提出意见。

一、2018年工作回顾

过去一年，是贯彻落实党的十九大精神开局之年。在市委的坚强领导下，在市人大、政协的监督支持下，我们以习近平新时代中国特色社会主义思想为指导，深入学习贯彻党的十九大和十九届二中、三中全会精神，认真贯彻落实习近平总书记考察内蒙古和参加十三届全国人大一次会议内蒙古代表团审议时的重要讲话精神，全面落实党中央重大决策部署，按照自治区党委、政府部署要求，坚持稳中求进工作总基调，践行新发展理念，团结依靠全市各族干部群众，同心协力，扎实工作，积极应对各种困难和风险挑战，实现了经济社会持续健康发展。地区生产总值完成1301.6亿元，增长4.1%。5000万元以上固定资产项目投资额增长13.5%。一般公共预算收入完成75.5亿元，增长7.1%。社会消费品零售总额完成563.7亿元，增长6.1%。城镇常住居民人均可支配收入达到31936元、增长7.6%，农村牧区常住居民人均可支配收入达到13797元、增长9.8%。

一年来，我们主要做了以下工作：

（一）提高政治站位，以实际行动践行“两个维护”

市政府始终坚持正确政治方向，树牢“四个意识”，坚定“四个自信”，坚决做到“两个维护”，在思想上政治上行动上同以习近平同志为核心的党中央保持高度一致。坚持把学习贯彻习近平新时代中国特色社会主义思想和党的十九大精神作为首要政治任务，把学习贯彻习近平总书记重要讲话、重要批示精神作为市政府重要会议首个议题，做到第一时间学习传达，第一时间研究落实。按照习近平总书记“四个着力”“三个扎实”重要要求，结合通辽实际，制定贯彻落实意见，持续推动习近平总书记重要讲话精神转化为通辽高质量发展具体实践。对标对表中央重大决策部署，一以贯之、持续用力抓好中央巡视、中央环保督察及“回头看”反馈问题和国家、自治区约谈检查事项整改工作，相继制定出台了绿色矿山建设规划、农牧民工工资支付管理办法等工作制度，以实际行动和工作成效诠释对党忠诚。

（二）聚焦聚力，精准施策，三大攻坚战有序推进

防范化解重大风险初战告捷。坚持把化解政府债务作为重点，严格规范政府举债行为，政府债务全部纳入预算管理。积极化解政府隐性债务，制定完善化债方案，压实化债举措，“十个全覆盖”10项工程内拖欠工程款全部化解，按时限要求完成年度政府债务化解任务。有序处置企业债券违约风险，开展非法集资、农村牧区高利贷等专项整治行动，全市金融风险总体可控。

脱贫攻坚取得阶段成果。集中开展脱贫攻坚专项整治行动。各级财政投入扶贫资金13.9亿元，全年减贫40819人，贫困发生率由2.87%降至1.4%，科左后旗、开鲁县、扎鲁特旗脱贫摘帽工作进入退出程序。全市90%以上的贫困人口实现产业链增收，脱贫户人均纯收入达到7383元，增长17.8%。实施京蒙合作项目40个，到位资金2.77亿元。为1.83万名贫困家庭学生发放教育补助资金6639万元，为4.85万建档立卡贫困人口免费健康体检。将符合条件的1.29万贫困人口纳入低保和特困人员保障范围。

污染防治初见成效。按时序进度完成14项中央环保督察反馈问题整改。北部地区电解铝烟气污染物超低排放示范工程建成运行。全年空气质量优良天数323天，同比增加7天。科尔沁超采区地下水位上升0.47米、缩小500平方公里，建成7座城镇再生水处理设施。开展土壤污染状况详查，农用化肥施用量同比下降10%以上，农药施用量同比下降4.3%。

（三）科学统筹，综合施策，经济发展稳中有进

供给侧结构性改革深入推进。关闭3座30万吨以下小煤矿。霍林河地区微网接入蒙西电网供电，蒙东电力公司取得区域内电厂调度运行管理权，通过电力多边交易和大用户直供，降低企业用电成本3.1亿元。为市场主体减轻税费负担23亿元。新通高铁开通运行，成为内蒙古首条接入全国高铁网的高速铁路。大沁他拉至白家湾子高速公路主线贯通。通辽机场改扩建工程获得国家民航局批复。毛都水库完成截流。

主导产业全链条发展。年产35万吨铝板带箔哈兹列特生产线建成投产，奈曼经安镍基材料项目正式开工，伯恩露笑1600台长晶炉全部投产，彩色蓝宝石研发成功填补国内空白。梅花生物苏氨酸、色氨酸项目正式投产，河南开药集团原料药项目建成试车。科尔沁牛业肉牛产业园10万头肉牛加工项目主体完工，伊赛20万头肉牛加工项目建成投产，全市肉牛加工能力达到60万头。康美药业、广州康臣等蒙医药合作项目顺利推进，蒙药股份、库伦蒙药被自治区列为蒙药配方剂、中药配方颗粒研发生产和临床使用试点。金煤化工聚乙醇酸项目中试取得初步成果。“九链五都”带动力、影响力持续提升。

农牧业现代化水平不断提升。土地规模流转面积达到407万亩，农业先进技术集成应用，粮食总产量163.3亿斤、增产13.1亿斤，道地药材种植面积达到27.7万亩。万头肉牛养殖场达到11个，优质肉牛存栏增长15.4%，我市被命名为“中国草原肉牛之都”，被认定为“科尔沁牛中国特色农产品优势区”。“通辽黄玉米”“科尔沁牛”分别获得全区农产品、畜产品区域公用品牌价值第一名。

新产业新技术新业态加快成长。编制《通辽市现代能源经济示范市建设规划》，被自治区列入“一区两基地”上报国务院。高新技术产业投资增长64.2%，国家级高新技术企业达到27家，霍林郭勒工业园区获评自治区级高新技术产业开发区。科尔沁牛业等3家企业获首届“市长质量奖”。电子商务交易额220.4亿元、增长20.2%，申通物流园项目一期投入使用。科尔沁500公里风景大道入选中国最受欢迎十大自驾路线，孟家段湿地旅游区晋升4A级旅游景区。成功举办第38届北京那达慕大会、首届通辽市肉牛文化产业博览会、全国民族医药高峰论坛暨内蒙古第四届蒙医药产业博览会和“一带一路”敖包相会系列活动。

（四）改革开放持续发力，发展体制机制逐步完善

重点领域改革取得积极进展。实施经济生态和社会事业领域改革举措120项。“放管服”改革持续推进，启动“最多跑一次”改革，推行投资项目并联审批、网上审批，市场主体达到20.4万户。完成霍煤集团2户所属企业混合所有制改革。紧密型农企利益联结比例达到60%。农村土地确权颁证、国有林场改革、农垦改革基本完成。83%的嘎查村和社区完成清产核资。农业水价综合改革取得积极进展。4个旗县农信社具备转制农商行条件。

对外开放水平不断提升。与蒙古国肯特省缔结友好城市，发运中欧（通满欧）双向班列12列。与吉林省四平市签署合作协议，与清华大学、吉林大学等校地合作取得实质性进展。柔性引进21名院士专家，组建通辽新型高端智库。在深圳、上海、杭州、武汉、沈阳等地举办招商引资项目推介会，全年签约亿元以上招商引资项目182个，到位资金183.2亿元。

（五）加大生态保护和修复力度，环境质量持续改善

“四个千万亩”工程顺利推进。坚持“北保护、中节水、南治沙”， 修复天然草原400万亩，启动建设北部现代畜牧业示范区，1080万亩草畜平衡区实行小畜全年禁牧、大畜半舍饲和划区轮牧；建成以浅埋滴灌为主的农业高效节水工程430万亩；实施沙化较重区域综合治理、生态廊道、“百万亩”经济林等工程，完成科尔沁沙地综合治理413万亩，库伦敖伦林场被命名为国家森林公园。

生态专项治理扎实开展。加大霍林河露天煤矿生态恢复治理力度，全面压实整改责任，科学制定治理方案，强化细化整改措施，企业投入治理资金1.4亿元。463个中央环保督察反馈的违法征占用草原项目整改全部完成。深入开展打击破坏森林资源违法犯罪专项行动，无立木林地恢复造林142万亩。

生态文明制度不断完善。落实最严格的耕地保护、水资源管理制度，“大棚房”问题清理整治取得阶段成果，完成水资源税费改革试点。全面落实河湖长制。实现重点企业和重点污染源 “三位一体”24小时自动监控和网格化执法监管全覆盖。完成领导干部自然资源资产任中审计试点。

（六）坚持以人民为中心的发展思想，民生福祉不断改善

文明城市创建成果持续巩固。深入推进“八大环境”建设，开展城市修补、生态修复，中心城区完成94处弃管换热站、86处老旧小区供热管网改造，加强中心城区餐厨垃圾收集运输处置管理，餐厨垃圾处理厂建成运行。城镇棚户区改造17484户，农村牧区危房改造5189户。完成农村牧区饮水安全巩固提升工程136处。扎实推进农村牧区人居环境整治，生活污水无害化处理覆盖1.5万户，卫生户厕普及2.5万户。

就业创业质量得到提升。实施10万大学生就业创业集聚计划，实现高校毕业生就业创业1.5万人。举办大型招聘推介活动83场，提升完善创业园区和孵化基地43家，实现城镇新增就业1.73万人，城镇登记失业率3.58%。开展失业保险援企稳岗“护航行动”，发放资金2112万元。推进退役军人安置，退役军人服务管理工作得到加强。

教育资源供给能力增强。多种形式补充教师515名。新增普惠性民办幼儿园28所，中心城区新增中小学3所。旗县义务教育均衡发展全部通过国家评估认定，基本消除起始年级“大班额”，校外培训机构治理工作初见成效。市工业职业学校投入使用，通辽职业学院获批设立蒙医学和口腔医学专业。

卫生健康服务不断改善。公立医院综合改革稳步推进，康美（通辽）医院合作项目、科尔沁区第四人民医院医养结合项目开工建设，14所公立医院和39所苏木乡镇卫生院开通远程医疗系统，15家医院实现跨省异地就医直接结算。第6次荣获“全国无偿献血先进市”称号。

文化体育事业加快发展。继承和发扬民族传统文化，加大非物质文化遗产保护力度。组织创办自治区级马头琴大赛等系列文化品牌赛事。全区乌兰牧骑东部区培训基地在通辽挂牌成立。通辽市全民健身中心主体完工，创新举办首届科尔沁运动大会，体育健身惠及城乡百姓。足球改革发展不断深入，取得多项赛事冠军。

社会保障水平稳步提高。企业职工、城乡居民养老金月人均分别增加134.5元和18元。城乡居民医保各级财政补贴提高到490元，基本医疗保险、大病保险、医疗救助、补充商业保险等多位一体的医疗保障体系进一步完善。城市最低生活保障标准和补助水平月人均分别达到670元、500元，农村最低生活保障标准和补助水平年人均分别达到5150元、3200元。加强医保基金监管，追缴违规资金1504万元。建筑领域农牧民工实名制全覆盖，合法权益得到保障。

公共安全形势稳定向好。平安通辽建设持续推进，安全生产事故起数和死亡人数分别下降33.3%和13.4%，交通事故死亡人数同比减少209人。一批多年遗留信访问题得到彻底化解。加强食品药品安全监管，完成“五小一场”升级改造1200家、占总数的50%以上。有效防控牛炭疽疫病，阻断非洲猪瘟疫情。深入开展扫黑除恶专项斗争，打掉黑社会性质组织4个、恶势力团伙100个、涉恶犯罪集团10个。31起现行命案全部侦破，刑事案件现案破案率达到60.3%，侦破积压10年以上命案10起，人民群众安全感进一步增强。

各项社会事业全面进步。民族团结进步创建活动深入开展，依法管理宗教事务。积极开展国防动员、国防教育、人民防空和拥军优属工作，支持军队建设，促进军政军民团结。气象地震、新闻出版、档案史志、外事侨务、妇女儿童、红十字会、慈善公益、科协、老龄、关心下一代和残疾人等事业取得新成效。

一年来，我们切实增强法治信仰和法治思维，依宪施政，依法行政，法治政府建设取得新进展。自觉接受人大及其常委会法律监督、工作监督和政协民主监督，378件人大代表建议和政协委员提案全部办复。认真履行党建、意识形态“一岗双责”，坚决执行中央八项规定精神，全面推进政府系统党风廉政建设和反腐败工作，坚决整治形式主义和官僚主义，干事创业、务实担当的氛围更加浓厚。

各位代表，过去一年的成绩，是在习近平新时代中国特色社会主义思想指引下，全市各族干部群众同心同德、奋力拼搏的结果。在此，我代表市人民政府，向全市各族人民，向所有关心支持通辽发展的同志们、朋友们，表示诚挚的感谢！

回顾一年来的工作，我们也清醒地认识到，一些目标任务完成的还不够理想，发展中仍面临着不少困难和问题，突出表现在：稳增长压力较大，缺少支撑性大项目，投资后劲乏力，消费需求不足；转方式、调结构任务艰巨，创新能力不强；财政收支矛盾突出，民生领域还存在短板，群众在教育、医疗等方面还有不满意的地方；多个领域不同程度存在风险隐患，风险意识和防控能力需要增强；政府职能转变还不到位，法治意识、服务意识有待提高，营商环境还需进一步改善。对于这些问题，我们要采取有力措施，切实加以解决。

二、2019年工作安排

今年是新中国成立70周年，是全面建成小康社会、实现第一个百年奋斗目标的关键之年。我们充分认识到，加快高质量发展其径已明，习近平总书记对内蒙古重要讲话、重要批示精神，为我们今后发展提供了政治遵循，指明了前进方向；加快高质量发展其时已至，国家支持东北全面全方位振兴，自治区支持东部盟市转型跨越发展，通辽迈入高铁时代，为我们扩大高水平开放、融入区域发展大格局，提供了难得机遇；加快高质量发展其基已奠，市委确定的发展思路和目标举措凝心聚力，新旧动能接续转换，发展环境持续改善，为我们全面建成小康社会，打下了坚实基础。只要我们把握主动，善作善成，认真办好自己的事，就一定能够开创现代化通辽新局面。

市政府工作的总体要求是：以习近平新时代中国特色社会主义思想为指导，全面贯彻党的十九大和十九届二中、三中全会精神，深入贯彻中央经济工作会议精神，全面落实自治区党委十届八次全会暨全区经济工作会议、自治区十三届二次人代会和市委五届七次全会暨全市经济工作会议精神，统筹推进“五位一体”总体布局，协调推进“四个全面”战略布局，坚持稳中求进工作总基调，坚持新发展理念，坚持推动高质量发展，坚持以供给侧结构性改革为主线，坚持深化市场化改革、扩大高水平开放，突出抓投资上项目，促进经济稳定增长；继续打好三大攻坚战，决战决胜全面建成小康社会；实施创新驱动发展战略，不断培育新的产业集群；持续优化营商环境，不断激发市场主体活力；实施乡村振兴战略，加快农牧业和农村牧区现代化；不断增进民生福祉，提升人民群众获得感、幸福感、安全感，统筹推进稳增长、促改革、调结构、惠民生、防风险工作，保持经济运行在合理区间，进一步落实“六稳”要求，确保经济持续健康发展和社会大局稳定，以优异成绩庆祝中华人民共和国成立70周年。

主要预期目标是：地区生产总值增长6%左右，限额以上固定资产投资增长10%左右，社会消费品零售总额增长7%左右，一般公共预算收入同口径增长7%，城乡常住居民人均可支配收入分别增长7%和9%，城镇登记失业率控制在4.5%以内，全面完成自治区下达的各项约束性指标。

为实现上述目标任务，着力做好以下工作：

（一）以重点战役为突破口，持续打好三大攻坚战。落实年度计划，坚决打赢重点战役，夺取决定性胜利。

防范化解重大风险。坚持底线思维，增强忧患意识，提高防控能力，完善防控机制，做实做细做好政治、经济、生态和社会等领域防范化解重大风险工作。稳妥化解政府债务，确保完成“十个全覆盖”10项工程外直接相关项目拖欠工程款化解任务，积极争取国家和自治区限额内专项债券规模，采取闲置资产变现、土地资源出让等措施，严格按时限要求完成年度化债任务。严厉打击非法集资、农村牧区高利贷和恶意逃废债行为，守住不发生系统性风险底线。

提升脱贫攻坚质量。认真抓好中央脱贫攻坚专项巡视反馈问题整改。各级财政计划投入扶贫资金14.6亿元，确保年内实现2.78万建档立卡贫困人口稳定脱贫，科左中旗、库伦旗、奈曼旗3个国贫旗达到脱贫摘帽标准。夯实产业脱贫基础，重点抓好肉牛养殖、特色种植等产业扶贫措施，让有劳动能力者有产业、无劳动能力者有分红，确保贫困人口增收15%以上。建立实施“七个一”工作机制，深化京蒙扶贫协作和中央单位定点扶贫。落实教育、健康扶贫举措，加快完善兜底保障政策体系，健全稳定脱贫长效机制。

持续推进污染防治。坚持生态优先，绿色发展，坚决打好蓝天、碧水、净土保卫战。实施电解铝烟气深度治理，重点企业持续减排并稳定达标排放。实施冬季清洁取暖计划，开展燃煤污染专项治理，严格按标准要求控建燃煤锅炉，燃煤发电机组全部实现超低排放，空气质量优良天数持续增加。深化地下水超采区综合治理，严厉查处超采、违规取用等行为。开展城镇、工业园区污水处理厂“三率”提升专项整治行动。各级河湖长常态化巡河巡湖，持续开展河湖“清四乱”专项行动。推进土壤污染治理与修复，实施畜禽粪污资源化利用整县推进项目，防控农业面源污染。加大中央环保督察及“回头看”反馈问题整改力度，重点抓好霍林河露天煤矿生态恢复治理。完善污染防治工作制度，严格落实环保“三同时”，严禁任何地区、任何行业以任何理由违反环保标准上项目。

（二）提升全产业链水平，夯实高质量发展基础。聚焦“九链五都”抓项目、强链条，推动质量变革、效率变革、动力变革，加快构建现代化产业体系。

以优质项目打造产业集群。实施500万元以上工业重点项目130个以上，完成工业固定资产投资170亿元以上，竣工、投产项目76个，规模以上工业增加值增长6.5%以上。加快建设铝镍硅新材料产业集群，推进锦联铝材、创源金属达产达效，联晟高精宽幅超薄铝箔、旭阳球形铝粉、经安镍基合金、蒙元碳材60万吨煅后焦、露笑150公斤级蓝宝石晶体等项目建成投产。加快建设玉米生物技术产业集群，开工建设梅花生物1万吨海藻糖、精晶药业1万吨氨基酸等项目，建成投产圣雪大成综合医药一期、华北制药生物发酵项目。加快建设肉牛产业集群，伊赛牛肉二期、科尔沁牛业肉牛产业园等项目建成投产，力争实现肉牛加工60万头。加快建设蒙医药产业集群，推动修正科尔沁药业与亿利健康资源合作，实施海天制药、天奈药业等蒙中医药合作项目，孟和制药项目建成投产。加快建设可降解新材料产业集群，实现聚乙醇酸规模化生产，可降解新材料产业园建设取得突破性进展。

大力发展新兴产业。积极创建现代能源经济示范市，加快建设蒙东能源负荷中心、清洁能源输出中心、电力交易中心。发展现代装备制造业，推进开发区红荣装配产业园、科尔沁区新天地电气低压箱柜等项目建设。发展数字经济，促进互联网、大数据、人工智能与制造业、农牧业、服务业深度融合。发展节能环保产业，科左中旗万众生物天然气、中心城区华通生活垃圾焚烧发电、科尔沁区海邻生物低碳饲料项目建成投产，开工建设北京三聚环保万吨级秸秆生物质综合利用项目，推动新兴产业上规模上水平。

发挥科技创新引领作用。深化与中科院、清华大学、吉林大学等科研院校合作，加强全产业链科技攻关。支持内蒙古民族大学建设蒙医药国家重点实验室和国家地方联合工程研究中心，提升内蒙古蒙医药工程技术研究院科研能力。建设内蒙古东部科学技术产权交易中心，推动科技成果引进转化。成立通辽市创新创业联盟，统筹协调全市双创资源，鼓励和引导企业建设科技创新平台，推进技术研发，开展质量提升行动，打造竞争新优势。

推动工业园区集约发展。完善工业园区规划布局，明晰产业定位，引导各类项目向园区集聚，彰显优势特色，实现错位发展。开展工业园区新一轮环评，加强工业园区环保基础设施建设，推进“三废”收集处理、固废渣场等功能性厂所建设，积极争取“无废城市”建设试点。优化工业园区资源配置，加快盘活土地等闲置资产。推进霍林郭勒工业园区和扎哈淖尔工业园区协同发展，加快扎鲁特旗、科左中旗同城工业园区整合，加快科尔沁工业园区东区搬迁改造，培育一批产值过百亿园区，争创自治区级和国家级高新技术产业开发区。

（三）实施乡村振兴战略，加快农牧业和农村牧区现代化。坚持农牧业和农村牧区优先发展，推动质量兴农、绿色兴农、品牌强农，建设乡村振兴现代农牧业示范区。

持续优化农牧业结构。压减籽粒玉米种植面积10万亩，种植经济作物260万亩，建设优质饲草料基地560万亩。加强耕地储备库建设管理，彻底清理整治“大棚房”问题，坚决遏制农地非农化。实施“稳羊增牛”战略，新增优质肉牛存栏10万头以上。确保通辽国家农业科技园区通过验收，争创国家农业高新技术产业示范区。推进无规定疫病区建设，持续抓好非洲猪瘟等动物疫病防控，保障畜牧业健康发展和肉食品安全。

大力发展品牌农牧业。实施千亿级农畜产品区域公用品牌创建行动，打造通辽绿色农畜产品“科尔沁”金字招牌。建立“三品一标”认证奖补机制，完善农畜产品标准体系和质量安全追溯体系，开展中国特色农产品优势区申报工作，提升地方区域公用品牌价值和影响力。

提高农牧业产业化水平。促进小农户与现代农牧业有机衔接，壮大农牧业产业化龙头企业，新增专业合作社150家，紧密型农企联结带动33万农牧户。健全新型农牧业社会化服务体系，发展多种形式的适度规模经营。加强智慧农牧业建设，拓展农牧业功能，促进一二三产业融合发展。⠼/span>

改善农村牧区人居环境。深入推进农村牧区人居环境整治，完成垃圾处理、污水处理、厕所革命等专项规划编制。启动村庄清洁行动，重点做好“三清一改”工作。新改扩建农村公路680公里，新增99个撤并建制村通沥青（水泥）路。加快推进农产品物流配送网络、宽带乡村等重点工程，健全完善农村牧区基础设施建设管护长效机制，巩固美丽乡村建设成果。

（四）推动服务业扩量提质，培育壮大发展新动能。适应高铁时代和消费升级新趋势，坚持生产性服务业与生活性服务业并重，推动现代服务业发展提速、比重提高、质量提升。

加快发展现代物流业。推进“两大基地”“六个中心”建设，打造集进口货物贸易、加工、集散中转为一体的中欧（通满欧）班列货物加工输出基地，启动通辽机场航空口岸临时开放申建工作，规划建设航空货运基地。力争建成运营通辽保税物流中心和多式联运海关监管中心。实施申通物流园二期项目，建设东北快递分拣中心。建成内蒙古蒙药材城，打造重要的蒙药研发销售和中蒙药材交易中心。构建线上交易、线下冷链配送的农畜产品交易模式，扩大农畜产品交易中心和肉牛交易中心成交规模。

加快培育新兴服务业态。提升文化、体育、旅游产业融合发展水平，培育千亿级文旅产业，启动实施机场、高铁落地自驾服务系统等项目，推动文旅发展与高铁时代相得益彰。开工建设通辽国际博览中心，举办国际蒙医药产业博览会、国际肉牛大会等展会活动，打造一批区域性、全国性会展品牌。引导和鼓励社会力量加大幸福产业投入，多形式增加健康、养老、育婴等服务供给，促进家政、快递、法律咨询、心理辅导等服务业加快发展。

（五）加大政策落实力度，发展壮大民营经济。精准有效落实促进民营经济高质量发展各项政策措施，切实解决民营企业实际困难，促进民营经济大发展。

支持民营企业做大做强。实施民营经济提升工程，力争新注册私营企业5000户。实施定点联系民营企业和商会组织制度，对困难民营企业进行“一对一”精准帮扶。加快建立现代企业制度，支持民营企业创新发展，实施高新技术企业倍增计划，促进民营企业“小升规”，加快培育一批具有引领支撑作用的大型民营企业。实施企业家素质提升工程，加强企业家队伍建设，弘扬工匠精神和企业家精神，培育一批优秀民营企业家。

扩大民营企业金融供给。设立民营企业流动性风险防控基金，为符合条件的民营企业提供转贷过桥服务。发挥“助保贷”作用，实施民营企业上市挂牌行动计划，鼓励引导企业用好多层次资本市场，扩大民营企业融资规模。强化担保能力建设，力争实现融资担保规模20亿元。

优化民营企业发展环境。开展“营商环境优化年”活动。加强政金企常态化、长效化合作。清理规范涉企收费，实施涉企行政事业性收费和政府性基金目录公示制度，确保清单外“零收费”。全面推行市场准入负面清单制度，推动“非禁即入”普遍落实。建立民营企业综合信息服务平台和民营经济发展工作联席会议制度，构建亲清新型政商关系。专项治理机关事业单位和国有企业拖欠民营企业资金问题，依法保护企业家合法权益。

（六）推进以人为核心的新型城镇化，建设宜居型城市。坚持规划先行、集约发展、精细化管理，促进城镇高质量发展，让人民群众在城市生活得更方便、更舒心、更美好。

提高规划管控能力。结合第三次国土调查成果，开展资源环境承载力和国土空间开发适应性“双评价”，开展城市总体规划实施评估，完成通辽市空间规划大纲编制工作。全面落实“多规合一”，科学划定城镇开发边界，发挥规划引领管控作用，增强城乡规划严肃性。优化城市公共设施布局，增加公共设施和公共服务用地供给。

完善中心城区服务功能。老城区以高铁站为中心，建设高铁经济圈，完成高铁站南广场主体工程，组织实施中心城区快速环线，启动大兴路至大广高速南出口道路工程，完成民主路和白音太来大街公铁立交桥引道、新世纪大桥连接线及互通式立交工程，红光大桥竣工通车，推进新工二路、福利路道路工程建设。加快改造老城区老旧供热管网、供水、排水、排污等市政设施，统筹建设供热管线互联互通及跨辽河等重点供热工程。新城区以行政办公区为中心，加快完善办公服务、商业商圈、教育医疗、体育休闲等服务功能，开工建设富力五星级酒店、亚琦集团通辽国际物流商贸城。加快内蒙古民族大学新校区建设，力争市科技馆、图书馆年内竣工投入使用。加快中心城区东出口改造，实施哲里木大桥至东外环路大桥段辽河河道治理工程。

高标准建设特色小镇。制定完善发展规划，集聚特色产业，集合文化、旅游、康养等功能，重点推进奈曼蒙中医药小镇、青龙山甘薯小镇、阿古拉 “一带一路·敖包相会”文化旅游主题小镇、舍伯吐肉牛小镇、东风红干椒小镇等特色镇建设，加快形成多样化城镇发展格局，以新型城镇化促进城乡融合，带动经济社会转型发展。

推进智慧城市建设。以“资源整合、数据汇合、应用融合”为核心，建设“平台通、数据通、应用通”智慧城市体系。华为、曙光云计算中心和视频信息共享平台投入使用，建成政务信息资源共享、智慧交通、智慧城管、智慧公安、智慧教育、智慧医疗、智慧社保、“一卡通”项目，让数据多跑路、群众少跑腿，用智能化推动生活便利化、服务现代化。⠼/span>

提升城镇管理水平。持续巩固“双城同创”成果，全面启动生活垃圾分类工作，开展“美丽街区”“无违建小区”“服务标兵”等创建活动，用绣花功夫管理城市。严格执行《通辽市城市市容和环境卫生管理条例》，坚持责权利统一、财权事权相匹配原则，理顺市区两级城市管理体制，切实提高城市精细化、法治化管理水平。

（七）深化改革开放，不断增强发展动力和活力。推动改革走深走实、开放登高望远，加快打造“一带一路”草原名城。

深化重点领域改革。以“一网一门一次”为重点，推动政务流程再造和信息资源共享，加快构建“互联网+政务服务”新模式，实现一般企业投资项目开工前审批“最多跑一次”“最多30天”办结。组建市国有资产监督管理委员会，实现经营性国有资产集中统一监管。加快政府融资平台实体化、市场化转型。巩固提升农村土地确权登记颁证、供销社综合改革、农垦改革、国有林场改革成果，落实土地“三权”分置改革政策，稳步推进农业水价综合改革。

扩大全方位对外开放。主动融入“一带一路”建设，深化同俄蒙合作，积极参加中国国际进口博览会、中蒙博览会、中蒙“3+3”区域合作会议等展洽活动。加快规划建设高铁经济带，全方位对接东北三省优势产业和高端要素，加快建设国家级东北振兴示范区。加强与东北亚、环渤海、京津冀、长三角、珠三角等地区的交往合作，积极承接产业转移。创新招商引资方式，压实市旗两级领导抓招商工作责任，完善招商项目全程跟进与服务评价机制，切实提高项目履约率、开工率、投产率。新签约亿元以上项目100个，新开工亿元以上项目100个。

（八）加大生态和基础设施建设力度，提升发展保障能力。聚焦关键领域和薄弱环节，补齐短板，增强后劲，提高绿色发展和可持续发展水平。

加强生态环境保护。积极争取并用好欧投行贷款、“三北”防护林工程等项目资金，继续实施“四个千万亩”生态修复保护工程，完成科尔沁沙地综合治理330万亩、天然草原修复300万亩、无立木林地恢复造林100万亩。设立矿山恢复治理基金，制定矿业权退出台账，落实治理修复方案，加快建设绿色矿山。加强自然保护区、湿地和草原保护，打击破坏森林草原和猎捕野生动物违法犯罪行为。严格落实国家重点生态功能区产业准入负面清单制度，严守生态保护红线、环境质量底线、资源利用上线，严禁不符合主体功能定位的各类开发活动。编制自然资源资产负债表，严格执行生态环境损害赔偿和责任追究制度，确保生态环境质量持续改善。

补齐基础设施短板。新改扩建国省干线公路700公里，大沁他拉至白家湾子高速公路、舍伯吐至哈根庙一级公路、鲁北至开鲁二级公路建成通车，完成京通铁路电气化改造，通辽机场飞行区改扩建工程建成使用，奈曼通用机场主体完工，通辽至霍林郭勒天然气管道建成运行，开工建设奈曼500千伏输变电工程，实施农网改造升级工程。加快储备一批基础设施领域补短板重大项目，推进通辽至齐齐哈尔、通辽至长春高速铁路、集通铁路电气化改造、甘库二期铁路、鲁北至霍林河高速公路、保康至舍伯吐一级公路前期工作。

提高水资源保障能力。完成农业高效节水工程年度任务，农田灌溉水利用系数达到0.64。力争LXB供水支线工程年内开工建设，加快“引绰济辽”二期项目前期工作。启动中心城区再生水利用规划，推动现有污水处理厂全部达标排放，在建污水处理、中水回用设施全部建成投用，中心城区污水处理和再生水利用率分别达到94%和50%以上。完成通辽发电总厂、通辽热电公司工业用水置换。强化饮用水水源地保护，完成集中式饮用水源地整治任务，三义堂水源地供水和通新水厂改扩建主体工程完工，巩固提升农村牧区8万人口饮水安全。

（九）持续保障和改善民生，努力满足人民群众美好生活需求。加强普惠性、基础性、兜底性民生建设，切实增强人民群众获得感、幸福感、安全感。

把稳就业摆在突出位置。完善就业创业扶持政策，实现城镇新增就业1.55万人。举办大中型招聘活动50场次以上，提供就业岗位3万个以上。抓好高校毕业生、退役军人等重点群体就业，实现1.75万名高校毕业生就业创业。打造劳务品牌，实现农牧民高质量转移就业46万人。实施高技能人才振兴计划，新增高技能人才1400人。

办好人民满意的教育。深化教育领域综合改革，加大教育投入力度，推动各级各类教育协调发展。加强新时代教师队伍建设，实施名教师、名校长培育工程，提高教师素养，弘扬高尚师德。推进学前教育普惠发展，大力发展公办园，积极扶持普惠性民办园，规范发展营利性民办园。巩固义务教育基本均衡发展成果，推进义务教育优质均衡发展，重点解决“择校选班热”、中小学生负担重等突出问题，常态化治理校外培训机构和“小饭桌”。促进民族教育内涵发展，加快通辽蒙中改扩建。推动普通高中优质特色发展，每个旗县市区办好一所示范性高中。提高职业教育质量，深化产教融合、校企合作。

实施“健康通辽”战略。深化公立医院综合改革，完善分级诊疗制度，提升基层诊疗水平和能力。深化京通等医疗卫生合作，扩大异地就医即时结算范围。完善医疗卫生行业综合监管制度，开展规范医疗服务行为专项整治。加强公共卫生服务体系建设，抓好重大传染病、地方病防治。实施蒙中医药服务能力提升工程，继续开展“名院名科名医”创建活动。推进药品安全监管和质量提升，创建国家食品安全示范城市。加快足球运动产业化步伐，启动建设内蒙古东部青少年足球训练中心。广泛开展全民健身运动，举办第二届科尔沁运动大会等大型群众体育赛事活动。

全面提升文化软实力。加快构建现代公共文化服务体系，推进基本公共文化服务标准化均等化。结合中华文明探源工程成果，启动《通辽史》编撰等系列跟进工作。扶持文化艺术精品创作。强化文化遗产保护和利用，大力扶持非遗项目及传承人，积极申建科尔沁文化生态保护区。创新发展民族优秀传统文化，举办国际乌力格尔论坛。

提高社会保障能力。持续扩大社会保险参保覆盖范围，健全完善社会救助体系，提高救助水平。加强和完善养老服务体系建设，鼓励各类社会资本进入养老市场，整合提质敬老院10所，通辽市老年人养护中心投入运营。完成棚户区改造、农村牧区危房改造年度任务。农牧民工工资支付保障制度基本实现全覆盖。

发展各项社会事业。深入开展民族团结进步创建活动，加强城市民族工作，积极创建全国民族团结进步示范市。做好退役军人服务管理工作，开展拥军优属活动，提高抚恤补助标准，构建军民融合发展新格局。争创全国双拥模范城，力争实现“五连冠”目标。坚持依法治统，做好第四次全国经济普查，开展新经济统计。加快通辽高分辨率气象分中心建设，实施市区两级气象局站整体迁建项目。加强国防动员、国防教育。提升人民防空、防震减灾工作水平。保障妇女儿童、未成年人权益，支持残联、红十字会等慈善公益事业发展。

加强和创新社会治理。全面做好医药卫生、食品安全、安全生产、建筑施工、道路交通等各方面工作，实现重大风险隐患排查全覆盖，落实风险防控责任，健全排查化解工作机制，强化源头治理，坚决遏制重特大事故发生。依法规范信访秩序，推动“事要解决”，下大气力解决好人民群众切身利益问题。深入实施“七五”普法规划，增强全民法治意识。深化“平安通辽”建设，创新完善立体化、信息化社会治安防控体系，纵深推进扫黑除恶专项斗争，严厉打击各类违法犯罪活动，持续压降交通事故亡人。加强应急管理体系建设，提升突发事件处置和综合救援能力。

（十）以政治建设为统领，切实加强政府自身建设。做好今年工作，各级政府必须把政治建设放在首位，切实增强“四个意识”，坚定“四个自信”，坚决做到“两个维护”，认真贯彻落实习近平新时代中国特色社会主义思想和党的十九大精神，确保党中央重大决策部署落地生根、开花结果。市政府作为“作战部”，要重实干、强执行、抓落实、作表率，努力干出无愧于时代和人民的新业绩。

建设服务型政府。切实把以人民为中心的发展思想融入到政府工作全过程，加强调查研究，深入基层，深入实际，深入群众，虚心听取各方面意见和建议，真正把群众在想什么、盼什么、需要我们干什么了解清楚，从高处看，向实处落，能为善为，亲力亲为，真正办好各项民生实事，以实实在在的工作成果造福全市人民。

建设法治政府。坚持依法行政，把政府工作全面纳入法治轨道，严格按法定权限履行职责，健全完善依法决策机制和政府法律顾问制度。自觉接受人大法律监督，主动接受政协民主监督，广泛接受社会监督和舆论监督，认真听取各民主党派、工商联、无党派人士和人民团体意见，保障人民群众知情权、参与权、表达权和监督权。

建设诚信政府。强化领导干部诚信意识，始终坚持实事求是，健全完善各项诚信制度，建立政务诚信监督和失信惩戒机制。加强招商引资、政府债务等重点领域政务诚信建设，依法依规履行政府承诺和各类合同协议，杜绝毁约失信，做到言必信、行必果。

建设高效政府。全面完成政府机构改革，按照“三定”方案，科学明岗定责，严肃编制人事管理，健全完善部门工作制度，切实履职尽责。面对新形势和新任务，我们要强化风险意识，树立底线思维，增强斗争精神，顶得住压力，打得了硬仗，善于化危为机。坚决破除形式主义、官僚主义，纠正凡事留痕的做法，让各级干部有更多时间和精力研究工作、推动落实、提高效能。

建设廉洁政府。全面加强政府系统党的建设，认真履行从严管党治党政治责任，严格落实中央八项规定精神和自治区党委、市委配套规定，驰而不息纠正“四风”。强化公共资金、国有资产、国有企业和领导干部经济责任的审计监督，发现问题坚决查处。树立过紧日子思想，厉行勤俭节约，大力压减一般性支出，确保“三公”经费只减不增。推进政务公开，让权力在阳光下运行。

各位代表，通辽已经迈入新时代，我们已经站上新起点。让我们更加紧密地团结在以习近平同志为核心的党中央周围，不忘初心，牢记使命，崇尚实干，埋头苦干，为全面建成小康社会、“建设亮丽内蒙古、共圆伟大中国梦”做出应有的贡献，以优异成绩庆祝中华人民共和国成立70周年！
